# Identification and *In Vitro* Reactivity of Celiac Immunoactive Peptides in an Apparent Gluten-Free Beer

**DOI:** 10.1371/journal.pone.0100917

**Published:** 2014-06-25

**Authors:** Ana Real, Isabel Comino, Mª de Lourdes Moreno, Miguel Ángel López-Casado, Pedro Lorite, Mª Isabel Torres, Ángel Cebolla, Carolina Sousa

**Affiliations:** 1 Departamento de Microbiología y Parasitología, Facultad de Farmacia, Universidad de Sevilla, Sevilla, Spain; 2 Departamento de Gastroenterología Pediátrica, Hospital Virgen de las Nieves, Granada, Spain; 3 Departamento de Biología Experimental, Campus Universitario Las Lagunillas, Jaén, Spain; 4 Biomedal S.L., Sevilla, Spain; Instutite of Agrochemistry and Food Technology, Spain

## Abstract

Gluten content from barley, rye, wheat and in certain oat varieties, must be avoid in individuals with celiac disease. In most of the Western countries, the level of gluten content in food to be considered as gluten-free products is below 20 parts per million measured by ELISA based on specific anti-gluten peptide antibody. However, in beverages or food suffering complex hydrolytic processes as beers, the relative proportion of reactive peptides for celiac patients and the analytical techniques may differ, because of the diversity of the resulting peptide populations after fermentations. A beer below 20 parts per million of gluten but yet detectable levels of gluten peptides by anti-gliadin 33-mer antibodies (G12 and A1) was analyzed. We identified and characterized the relevant peptides for either antibody recognition or immunoactivity in celiac patients. The beer was fractionated by HPLC. The relative reactivity of the different HPLC fractions to the G12/A1 antibodies correlated to the reactivity of peripheral blood mononuclear cells isolated from 14 celiac individuals. Peptides from representative fractions classified according to the relative reactivity to G12/A1 antibodies were identified by mass spectrometry. The beer peptides containing sequences with similarity to those of previously described G12 and A1 epitopes were synthesized and confirmed significant reactivity for the antibodies. The most reactive peptides for G12/A1 also confirmed the highest immunogenicity by peripheral blood mononuclear cell activation and interferon γ production from celiac patients. We concluded that preparative HPLC combined with anti-gliadin 33-mer G12/A1 antibodies were very sensitive and specific methods to analyze the relevant immunogenic peptides in hydrolyzed gluten.

## Introduction

Celiac disease (CD) is the most common food intolerance in Western countries, with an estimated prevalence that may rise up to 1% in the Caucasian population [1]. CD can be considered as the intolerance of genetically predisposed individuals to gluten polypeptides from wheat, rye, barley and to lower extend, certain oat varieties [2]–[4]. Gluten is a complex of storage proteins that contains high amounts of the amino acids glutamine, glutamic acid and proline [5]. As a consequence, these proteins are poorly degraded by the gastrointestinal enzymes and remain as relatively large peptides when entering the small intestine. The ability of gluten proteins to resist degradation was suggested to be one reason for their harmful effect on susceptible people [6]. In celiac individuals, immunogenic gluten peptides are deamidated by tissue transglutaminase which association generates potent autoantigens. These biochemical interactions elicit a T-cell mediated pathological response which consequences are the lymphocytary infiltration of the intestinal epithelia and the destruction of intestinal villi. This last effect makes CD patients to suffer from malabsorption and malnutrition that may lead to diarrhea, constipation, iron-deficiency anemia, osteoporosis, dermatitis herpetiformis and even neurological disorders [7]–[9].

The only treatment for CD is a life-long strict gluten-free diet (GFD), which normally leads to a complete remission of the symptoms and mucosal histology [1]. However, a GFD is difficult to maintain since this is a very common food additive. National public organizations and international institutions, as Current Codex Alimentarius and Food and Drug Administration (FDA), propose immunological methods based on antibodies against specific gluten peptides as feasible and reliable methods to ensure the absence of gluten from barley, wheat and rye in food and beverages [10], [11]. The use of antibodies specific to epitopes directly associated to the immunogenicity of the gluten peptides may reduce those cases of underestimation or overestimation of relevant gluten peptide content. Methods based on the antibodies for the immunogenic 33-mer peptide -G12 and A1- have been accumulating evidences for the detection of the dominant gluten immunogenic peptides for celiac patients in the food [2], [12]–[14]. Hydrolyzed gluten food or beverages could be the kind of samples where the measured gluten content could mostly differ from the celiac immunogenicity depending on the target sequences to be detected.

In a recent report, we examined the levels of gluten peptides equivalent to one of the most immunoactive protease-resistant gliadin 33-mer in 100 Belgian beers, using immunochromatographic (IC) lateral flow test with G12 and A1 antibodies [14]. The G12/A1 reactivity of beer HPLC fractions correlated to the presence of previously described T-cell reactive epitopes. In order to characterize low abundant reactive beer peptides to G12/A1 immunological methods, we have examined a beer legally classified as ‘gluten-free’ because the net content of gluten was below 20 parts per million (ppm) but with detectable levels indicating trace quantities. We have identified and determined the relevance of the immunoactive peptides in beer detectable by G12/A1 IC-strips and G12 competitive ELISA. We have sequenced peptides in HPLC fractions enriched in reactivity to G12/A1 IC-strips and some of them synthesized. The most reactive peptides to G12/A1 also showed the highest reactivity to peripheral blood mononuclear cells (PBMCs) proliferation and interferon γ (INF-γ) production from celiac patients.

## Materials and Methods

### Anti-gliadin 33-mer moAbs and peptides

Horseradish peroxidase (HRP) conjugated G12 and A1 moAbs (G12-HRP and A1-HRP) and the 33-mer peptide were obtained from Biomedal SL (Sevilla, Spain). The peptides PP 24.1 (PQPQQPQLPFPQQPQQPFPQPQQP), PP 24.2 (PQPQLPFPQQPQQPFPQPQQPQQP), PP 24.3 (PQPQQPQLPFPQQPFPQPQQPQQP), PP 22.1 (PQQPQLPFPQQPFPQPQQPQQP), QP 22.2 (QPQQPFPLQPQQPFPQQPPQQP), QP 7.1 (QLPYPQP), QP 7.2 (QQPFPQP), QQ 7.3 (QLPFPQQ), QQ 7.4 (QQPFPQQ), QQ 7.5 (QQPFPLQ), QY 6.1 (QPQLPY), QF 6.2 (QPQLPF), QF 6.3 (QPQQPF), and QQ 6.4 (QPQQPQ) were supplied by Biomedal SL (Sevilla, Spain).

### Beer protein fractions

Sample barley beer (type Strong Ale) was extracted using UGES according to the instructions of the manufacturer (Biomedal SL, Sevilla, Spain). Beer was separated into different fractions by reversed-phase HPLC (RP-HPLC) on a semi-preparative C_18_ column and, next, each fraction was separated by RP-HPLC on an analytical C_18_ column [14].

### MoAbs G12/A1 immunochromatographic test

The assay was carried out with the GlutenTox sticks kit according to the instructions of the manufacturer (Biomedal SL, Sevilla, Spain). Beer fractions were diluted (1∶10 to 1∶300) in the buffer solution provided and the gluten content was tested. The test sticks were dipped into the solution (300 µl) for 10 min before being removed and allowed to air dry.

### Enzyme-linked immunosorbent assay

Maxisorp microtitre plates (Nunc, Roskilde, Denmark) were coated with gliadin solution (Sigma, St Louis, MO, USA) and incubated overnight at 4°C. The plates were washed with phosphate-buffered saline (PBS) containing 0.05% Tween 20 and blocked with 5% non-fat dry milk in PBS for 1 h at room temperature (RT). 33-mer peptide was used as standard. Serial dilutions of peptides were made in PBS-bovine serum albumin (BSA) 3%, to each of which was added G12-HRP or A1-HRP antibody solution [14]. The samples were pre-incubated at RT, and then added to the wells. After 30 min of incubation at RT, the plates were washed and substrate solution (TMB, Sigma) was added. The reaction was stopped with 1 M sulphuric acid, and the absorbance at 450 nm was measured (microplate reader UVM340; Asys Hitech GmbH, Eugendorf, Austria). Two separate assays were performed, each with three repetitions.

### Mass spectrometry

Beer fractions were analysed by nano LC-Electrospray Ion Trap Mass Spectrometry (LC-ESI-IT-MS) (Ultimate 3000 nano HPLC, Dionex, Sunnyvale, California, USA) and HCT Ultra ion-trap Mass Spectrometry (Bruker Daltonics, Bremen, Germany). For each fraction, 2 µg of total protein was reconstituted in nano HPLC loading buffer (98% H_2_O milli-Q +2% acetonitrile +0.05% TFA). The flow rate was 30 µl/min and the injection volume was 5 µl. Absorbance was monitored with the UV-visible detector at 214 nm and 280 nm. Eluting buffers were buffer A (H_2_0 milli-Q +0.1% formic acid) and buffer B (80% acetonitrile +20% H_2_0 milli-Q +0.1% formic acid). Proteins were eluted by applying the following gradient conditions: isocratic elution with 4% B for 5 min; 4% to 40% B for 60 min; 40% to 95% B for 1 min; and isocratic elution with 95% B for 7 min. Mass spectrometric data were acquired in the automated data-dependent mode. Mass- and charge-dependent collision energies were used for peptide fragmentation. The 4 most-abundant ions were isolated and fragmented using collision-induced dissociation (CID) (4s/MS/MS spectrum). The spectra obtained were processed using DataAnalysis 3.4 software (Bruker Daltonics, Bremen, Germany) for analysis of raw data. Peptide masses obtained were exported to BioTools 3.1 software (Bruker Daltonics, Bremen, Germany), and the identification of proteins was carried out by searching for Viridiplantae taxonomically restricted in the database of the National Center for Biotechnology Information (NCBI), using Mascot v.2.3.02 (www.matrixscience.com, Matrix Science, London, UK).

### Histological and serological analysis of subjects

Fourteen patients with biopsy-proven active CD were included in this study. The diagnosis of CD was based on the positive serology and compatible lesion in the duodenal biopsy according to the criteria of Marsh [15] and confirmation of a clinical response to gluten elimination from the diet. Subjects were prospectively screened for CD using anti-endomysial antibodies (AAEMs), tissue transglutaminase antibodies (AATGs) and CD-specific human leukocyte antigen typing (HLA-DQ) (**Table 1**). Venous blood was taken at the time of index biopsy. The international standards for ethics (Helsinki declaration for studies in humans) were followed and the study was approved by the ethics committee of the ‘Virgen de las Nieves’ Hospital, Granada (Spain). The written informed consent was obtained from the next of kin or guardians on behalf of the minors enrolled in this study.

**Table 1:** Clinical data of patients with Celiac disease.

Patient	Age (year)	Sex	Weight (Kg)	Height (cm)	AATG (IgA)	AAEM	Atrophy grade (Marsh criteria)	HLA-DQB1
**Celiac 1**	4	Female	20	106	>200	+	IV	0201-0202
**Celiac 2**	4	Female	17.5	108	>200	+	III C	0201-0202
**Celiac 3**	1	Female	7.5	76	142	+	III B	N.D.
**Celiac 4**	3	Female	11	90	34	+	III A	0201-0603
**Celiac 5**	12	Male	49	151	>200	+	IV	0201-0503
**Celiac 6**	7	Male	23	123	>128	+	III A	0201-0301
**Celiac 7**	1	Male	10.5	82	>128	+	II	0201-0602
**Celiac 8**	5	Female	19	108	>128	+	III A	0201-0501
**Celiac 9**	10	Male	23.5	129	>200	+	IV	0201-0301
**Celiac 10**	2	Female	14	93	>128	+	III B	N.D.
**Celiac 11**	10	Female	24	132	>128	+	III B	0201-0202
**Celiac 12**	2	Female	13	92	16	+	III A	N.D.
**Celiac 13**	3	Male	13.5	91	91	+	III C	0201-0604
**Celiac 14**	5	Male	17.5	106	>200	+	III C	0201-0202

AAEM: antiendomysial antibody; AATG: antitransglutaminase antibody, expressed as U/ml; HLA: human leukocyte antigen; N.D. no data.

### Cell proliferation analysis

The alcohol-soluble protein fractions were extracted from wheat (*Triticum aestivum*) and rice (*Oryza sativa*) whole flour, positive and negative control, respectively. These fractions were subjected to pepsin, trypsin and chymotrypsin sequential digestion, as previously described by Real et al [3].

PBMCs which include lymphocytes (T cells, B cells and NK cells), monocytes and dendritic cells, from patients with active CD (n = 14) on gluten-containing diet were isolated from 6 ml of heparinized blood by Histopaque gradient centrifugation, and cultured at a density of 1x10^6^ cells/ml in RPMI-1640 culture medium. After 48 h, PBMCs were incubated with the different samples and the proliferation was determined using a non isotopic assay based on the incorporation of 5-bromo-2-deoxyuridine (BrdU) into the newly synthesized DNA according to manufacturer's instruction (Millipore Chemicon, Temecula, California, USA) [16], [17]. Briefly, once the media containing BrdU is removed, the cells are fixed and the DNA is denature. Then an anti-BrdU mouse monoclonal antibody is added followed by and HRP conjugated secondary antibody. Cells without BrdU reagent added (background) and only tissue culture supernatant (blank) were used as controls. The stimulation index (SI) value was calculated by dividing the mean absorbance/10 at 450 nm after stimulation by the mean absorbance of PBMCs exposed to the culture medium alone (background) and divided by 10. All cultures were performed in duplicate.

### IFN-γ production

Supernatants from PBMC culture were collected after 48 h and stored at −80°C for IFN-γ determination using a commercial ELISA kit in accordance with the manufacturer's instructions (Thermo Scientific, Madrid, Spain). Standards were run on each plate. Assay sensitivity was less than 2 pg/ml. All cultures were performed in duplicate.

### Statistical analysis

#### Anti-gliadin 33-mer ELISA

Peptide curves were obtained by plotting percentage maximum absorbance against logarithm of antigen concentration. The software package Sigma Plot 9.0 (Systat Software, Inc., Point Richmond, CA, USA) was used to calculate IC_50_ and the cross-reactivity (CR) for each peptide. The IC_50_ is defined as the concentration that produces a reduction of 50% in the peak signal in the ELISA. The CR was determined as (IC_50_ of the peptide that presents the greatest affinity for the antibody/IC_50_ of each peptide assayed) x 100.

#### Cell proliferation and IFN-γ assays

Statistical analysis was performed with the STATGRAPHICS program. Data are expressed as mean ± SD. When the interaction was statistically significant, the differences between groups were examined by one-factor analysis of variance (ANOVA). A Bonferroni-corrected *t*-test was used to compare the individual means. A statistical probability of *p*<0.05 was considered significant.

## Results and Discussion

### Immunoactive potential determination of beer fractions

Barley and wheat are the main cereals used in the production of malt beer, the first step in the brewing. More than 40 different proteases in addition to amylases are activated by malting. The metabolic barley proteins that are released in the water solution during mashing along with a much lower amount of hordeins, are extensively hydrolyzed by the endogenous proteolytic enzymes into short peptide fragments [18], [19]. Due to their high content in proline and glutamine, a heterogeneous mixture of peptides could be resistant to proteolysis that could be toxic for CD patients.

Previous proteomic investigations support a strict consistence of the beer proteome regardless of the brand under a qualitative standpoint, while detectable differences appear confined to the relative quantitative balance among protein components [20]. The capacity to characterize a beer with low gluten peptide content could then be expanded to any beer with superior concentration. A Belgian commercial beer (Strong Ale) classified as potential ‘gluten-free’ (<20 ppm gluten) according to normative of Codex Alimentarius, was selected as a representative sample for the extensive immunological analysis [14]. To that purpose, this beer was fractionated by RP-HPLC on a semi-preparative C18 column and, next, each fraction was separated by RP-HPLC on an analytical C18 column and assessed by G12/A1 IC-strips. This beer presented 6 reactive fractions out of 18 total fractions, all of them eluted at retention times *>*10.5 min [14]. Therefore, three groups of fractions could be distinguished: a group with reactivity >500-fold above the detection limit (E16 and E17), a group with intermediate reactivity (E13-E15 and E18) and another group comprising fractions that were not recognized by moAbs (E1-E12). Taking into account that the range of gluten content was about 10 ppm by competitive G12 ELISA, the RP-HPLC technique allowed to concentrate about 50-fold the reactive peptides in E16 and E17 fractions.

**Figure 1:**
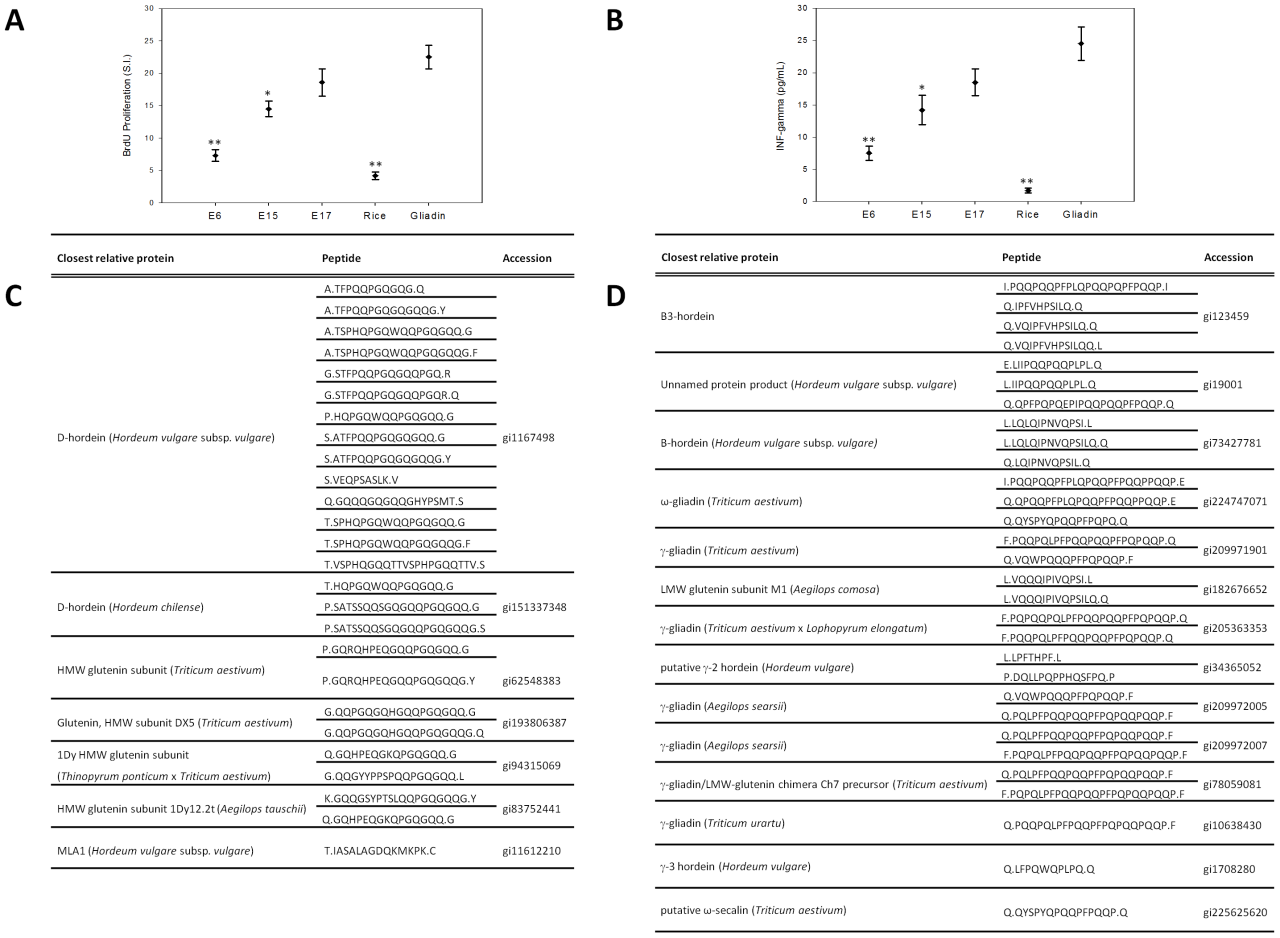
Immunogenicity of different elutions obtained by HPLC. **A.** Proliferative responses of PBMCs to three different elutions. **B**. IFN-γ production by PBMCs with three different elutions. In A and B, the results are expressed as mean ± SD of duplicated cultures (n = 14). The significantly differences with respect to gliadin were found at *p<0.05 and **p<0.005. Gliadin and rice prolamins were used as the positive and negative controls, respectively. **C** and **D**. Peptide fragment identification for E6 and E17 respectively, by Mascot after mass spectrometry.

To test the correlation in G12/A1 reactivity and the immunogenicity of the different fractions *in vitro*, three of them were selected based on the different level of reactivity to G12/A1 moAbs and were assessed by PBMC proliferation and IFN-γ response from peripheral blood isolated from celiac patients. The clinical and immunological profiles of patients with CD are presented in **Table 1**. Fraction E6 was chosen as representative of non-reactive group, E15 represented the group with intermediate reactivity and E17 that of the greatest reactivity. We found significant differences in PBMCs proliferation with respect to gliadin (positive control) in cultures incubated with E6 (SI = 7.3±0.9), which showed an activation of PBMCs slightly higher than rice prolamins (negative control SI = 4.2±0.6) (**Figure 1A**). E17 was the most reactive to G12/A1 IC-strips and showed the highest increase in PBMCs proliferation (SI = 18.6±2.1), and did not show significant differences with respect to cultures incubated with positive control (SI = 20.1±1.8). We found intermediate proliferation in cultures incubated with E15 (SI = 14.5±1.2) (**Figure 1A**).

Release of IFN-γ in the culture medium after the exposure of celiac PBMCs to the different representative fractions was assessed (**Figure 1B**). The highest values of IFN-γ release were found in supernatant of culture incubated with E17 (18.5±2.1 pg/ml) close to the gliadin values (22.5±2.6 pg/ml). We found significant differences with respect to positive control in cultures exposed to E6, which induced a lower mean value of IFN-γ (7.5±1.02 pg/ml).

We selected one reactive fraction (E17) and one non-reactive fraction (E6) in order to carry out the protein identification using Mascot after mass spectrometry. The comprehensive list of peptide fragments identified in the E6 and E17 fractions is shown in **Figures 1C and 1D**, respectively. These dates confirm that the sample beer contained a large number of partially degraded fragments from the gluten proteome of barley and wheat. A total of 7 different gluten proteins were identified in E6 and 14 in E17. The peptides identified differed in both their amino acid composition and length. It was interesting to know that in the E17 fraction, the hordein and gliadin derived peptides identified showed several motifs associated with the induction of CD. Among them, we could find the ‘PQQPF’ sequence, described as one of the main gliadin toxic motifs [21]. In that way, T-cell epitopes as ‘FPQQPQQPF’, ‘QQPQQPFPQ’, ‘QLPFPQQPQ’ and ‘QQPFPQQPQ’ were identified in different peptides present in E17 but not in E6 [22]–[25].

Beers with gluten peptide content close to the quantitation limit detected by the current analytical methods by antibodies (classified according to international labelling rules as ‘gluten-free foods’, <20 ppm, for Codex Alimentarius and/or FDA), should be analyzed in combination with *in vitro* immunological methods as T-cell proliferative response.

### Characterization of relative 33-mer epitopes in MS beer peptides

**Figure 2:**
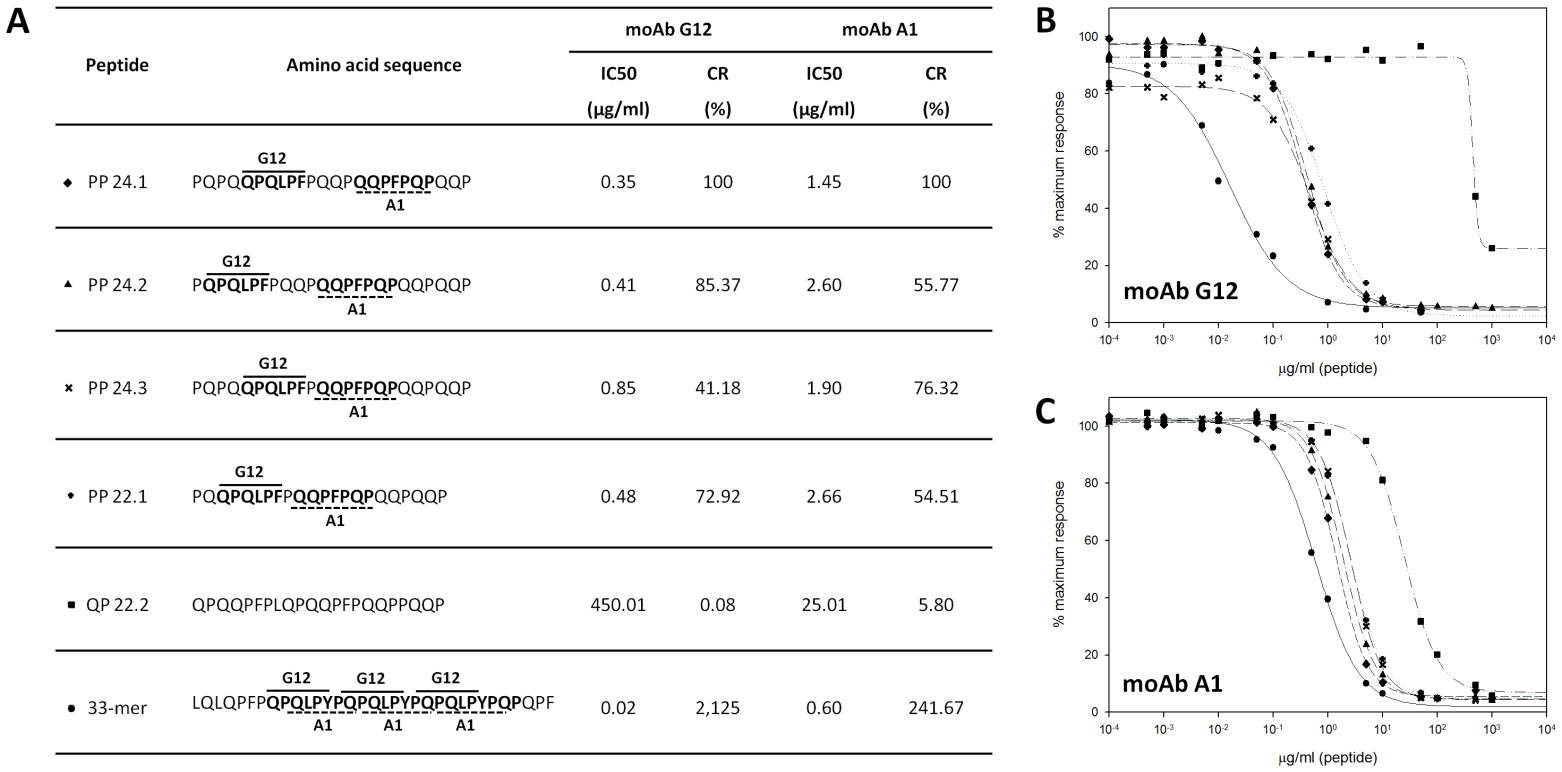
Relative affinity of the anti-gliadin 33-mer monoclonal antibodies for different peptides identified. **A.** Amino acid sequences of the peptides synthesized. IC_50_ and CR were obtained by G12 or A1 competitive ELISA. Epitope recognition of G12 and A1 moAbs are indicated by continuous and dashed line respectively. **B** and **C**. Competitive ELISA to determine the relative affinity of these antibodies for the different peptides. Two assays were performed, with three replicates of each.

Different research lines have shown that the reactivity of moAbs G12 and A1 is correlated with the real potential immunotoxicity T-cell-reactivity analysis and enzymatic detoxification of the proteins showed that the signal of these antibodies was correlated with the potential toxicity of the sample for celiac patients [2], [13], [14], [26], [27]. Many of the peptides identified in E17 contained runs Gln and Pro that may elicit an immunological response in celiac patients. To confirm the capacity of the suspected peptides of E17 fraction to be detectable by G12/A1 and their immunogenicity for celiac, we chose different peptides with similarities to epitopes for G12 (QPQ(L/Q)P(Y/F/Q)) and A1 (Q(L/Q)P(F/Y)P(Q/L)(P/Q). A total of five peptides of the sequenced fragments with length of 22 or 24 amino acids presented in the E17 were synthesized (**Figure 2A**). The affinity of the moAbs to different peptides was determined by competitive ELISA using a standard curve of the gliadin 33-mer peptide, the main immunodominant toxic peptide in celiac patients and one of the digestion-resistant gluten peptides [6]. The IC_50_ and CR were determined for each peptide. As shown in **Figure 2A**, PP 24.1 peptide presented the greatest reactivity for G12/A1 antibodies. Peptides PP 24.2, PP 24.3 and PP 22.1, were also recognized with high sensitivity by G12 and A1. The peptide QP 22.2 showed a drastic decrease in affinity for G12 and A1 moAbs (CR<6% for both antibodies).

Currently, the known antibodies recognition sequences described in toxic cereal sequences included nine different heptapeptides for A1 moAb, and five hexapeptides for G12 moAb [12]. Two epitopes of those 14 total sequences were found in the identified beer peptides. Specifically, we detected the highly reactive epitope sequences for G12 and A1, QQPFPQP and QPQLPF respectively (**Table 2**) in the most reactive peptides: PP 24.1, PP 24.2, PP 24.3 and PP 22.1 (**Figure 2A**). However, the QP 22.2 peptide did not show any of these G12/A1 previously described epitopes (**Figure 2A**), which may explain the poor reactivity for A1 and G12 antibodies.

**Table 2:** Relative affinity of anti-gliadin 33-mer moAbs for different peptide variants derived from its recognition regions.

A1 moAb			G12 moAb		
Peptides	IC50 (µg/ml)	CR (%)	Peptides	IC50 (µg/ml)	CR (%)
**QLPYPQP**	0.66	100.00	**QPQLPY**	0.23	100.00
**QQPFPQP**	0.17	388.23	**QPQLPF**	0.92	25.00
QLPFPQQ	11.79	5.59	QPQQPF	70.22	0.32
QQPFPQQ	32.08	2.05	QPQQPQ	N.A	N.A
QQPFPLQ	26.86	2.45			

IC_50_ values of the antibodies to peptides are indicated. N.A.: Not applicable. Antibody epitopes described are in bold and amino acids modified are in grey and underlined.

We identified potential variants epitopes recognized for these antibodies contained in these MS sequenced peptides. Thus, we found two variants derived from the G12 epitope QPQLPF and three derived from A1 epitope QQPFPQP with one or two modifications in the amino acid sequences (**Table 2**). To determine the relative affinity of the anti-gliadin 33-mer antibodies for these potential epitopes present in beer peptides described, we constructed hexa and heptapeptide variants of the G12 and A1 epitopes, respectively. The affinity of the anti-gliadin 33-mer moAbs for different variants was determined by competitive ELISA with immobilized gliadin in microtiter wells and was challenged with the synthetized peptides (**Table 2**). For A1 moAb, we found one different variant in the MS high reactive peptides (PP 24.1, PP 24.2, PP 24.3 and PP 22.1), in which, a proline was replacement by glutamine in the last position (QLPFPQQ), showing CR of 5.59%. We found other variants of the A1 recognition sequence, QQPFPQQ and QQPFPLQ, contained in QP 22.2 peptide that presented 30-fold lower affinity to this antibody than those ones of the epitopes located in the high affinity peptides. The variants studied for G12 moAb showed a dramatic reduction in affinity. Therefore, three new reactive sequences were identified for A1: QLPFPQQ, QQPFPLQ and QQPFPQQ. A new sequence QPQQPF showed to be slightly recognized by G12. In any case, there was a correlation between the epitope sequence of the peptides and the reactivity of the anti-gliadin 33-mer moAbs for the different peptides from the MS sequencing.

### Immune stimulatory properties of the beer peptides

**Figure 3:**
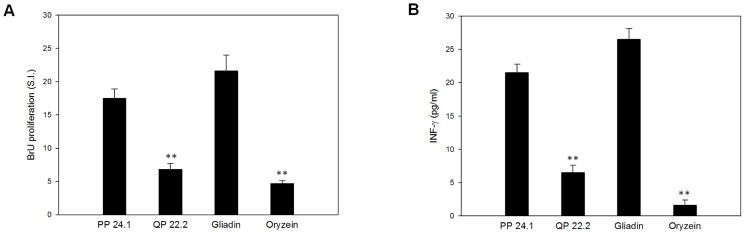
Immunoactive potential of peptides identified. **A.** Proliferative responses of PBMCs to different peptides. **B**. IFN-γ production by PBMCs with different peptides. In A and B, the results are expressed as mean ± SD of duplicated cultures (n  = 14). Gliadin and oryzein were used as the positive and negative control, respectively, and significantly different with respect to gliadin at **p<0.005 are shown.

To determine whether the variations in the reactivity of anti-gladin 33-mer antibodies towards the different peptides were correlated with the immunogenicity of peptides, we determined PBMCs stimulatory activity. Two peptides were selected, the most reactivity peptide (PP 24.1) and the peptide with the least affinity (QP 22.2) according to results obtained with the antibodies. The stimulatory activity of peptides was determined by PBMC proliferation and IFN-γ production. The ability of these peptides to induce a noxious immune response was studied in comparison with wheat gliadin and rice prolamine as positive and negative control, respectively. The results of cell proliferation from celiac PBMCs clearly showed that the peptide QP 22.2 induced a weak proliferative response (SI = 6.8±0.9), slightly superior to negative control (SI = 4.7±0.4). Thus, we found significant differences with regards to the cultures incubated with gliadin (SI = 21.6±2.4) (**Figure 3A**). We could observe similar results in IFN-γ production assays, where the exposure with the peptide QP 22.2 resulted in a lower IFN-γ response (6.5±1.1 pg/ml) with respect to high production by cultures incubated with gliadin (26.5±1.6 pg/ml). In both assays, the peptide PP 24.1 was the most immunogenic with an increase of cell proliferation and IFN-γ production (17.5±1.4 and 21.5±1.3 pg/ml, respectively) close to those the positive control (21.6±2.4 and 26.5±1.6 pg/ml, respectively) (**Figure 3B**). These results were consistent with those earlier obtained by competitive ELISA using anti-gliadin 33-mer antibodies. In fact, the peptide PP 24.1 was the most potentially immunoactive according to antibodies and cell proliferation. Thus, a direct correlation of the moAbs reactivity and the immunogenicity of peptides were corroborated.

We observed that the peptides recognized with the highest affinity by G12/A1 moAbs showed previously described T-cell epitopes in their sequences [25]. However, the peptide recognized with the lowest affinity by G12 and A1 moAbs (QP 22.1), did not show any T-cell epitopes described to date. Vader et al. (2002) found a frequently response to nondeamidated gluten peptides, and suggested a model where the deamidation is not a prerequisite for the initiation of response [28]. In this work, we have shown by *in vitro* studies a direct correlation of the immunogenicity of the different nondeamidated beer peptides with the considerable variability toxicity of peptides present in beer. We have examined that *in vitro* analysis may enable identification, selection, or production of different beer fractions with low levels of noxious gluten proteins.

In any case, the *in vitro* reactivity to PBMCs of celiac patients showed a good correlation to the reactivity of immunological methods with the anti-gliadin 33-mer G12/A1 antibodies. Although the diversity of peptide populations in hydrolyzed food containing immunogenic cereals could be unlimited, the reactivity of current methods with G12/A1 antibodies seems to react quantitatively with those remaining peptides with potential immunogenicity.

Future clinical studies with celiac patients would be necessary to know which level of hydrolyzed gluten content in beer provides reasonable safety. The underestimation of toxic gluten by those antibodies not discriminating the immunoactivity of the peptide might suppose an accumulative damage for celiac safety.
